# Urinary Exosome-Derived microRNAs Reflecting the Changes in Renal Function in Cats

**DOI:** 10.3389/fvets.2018.00289

**Published:** 2018-11-20

**Authors:** Osamu Ichii, Hiroshi Ohta, Taro Horino, Teppei Nakamura, Marina Hosotani, Tatsuya Mizoguchi, Keitaro Morishita, Kensuke Nakamura, Noboru Sasaki, Mitsuyoshi Takiguchi, Ryo Sato, Kazuhisa Oyamada, Yaser Hosny Ali Elewa, Yasuhiro Kon

**Affiliations:** ^1^Laboratory of Anatomy, Department of Basic Veterinary Sciences, Faculty of Veterinary Medicine, Hokkaido University, Sapporo, Japan; ^2^Laboratory of Veterinary Internal Medicine, Department of Veterinary Clinical Sciences, Faculty of Veterinary Medicine, Hokkaido University, Sapporo, Japan; ^3^Department of Endocrinology, Metabolism and Nephrology, Kochi Medical School, Kochi University, Nankoku, Japan; ^4^Section of Biological Safety Research, Chitose Laboratory, Japan Food Research Laboratories, Chitose, Japan; ^5^Veterinary Teaching Hospital, Faculty of Veterinary Medicine, Hokkaido University, Sapporo, Japan; ^6^Organization for Promotion of Tenure Track, University of Miyazaki, Miyazaki, Japan; ^7^Matsubara Animal Hospital, Matsubara, Japan; ^8^Department of Histology and Cytology, Faculty of Veterinary Medicine, Zagazig University, Zagazig, Egypt

**Keywords:** urinary exosome-derived miRNA, cats, kidney disease, biomarker, next-generation sequencing

## Abstract

Increased incidence of kidney disease (KD) is a common concern in human and companion animals. Cats, in particular, are highly susceptible to KD. Novel KD biomarkers would help to address these problems. Therefore, we are focusing on microRNA, a highly conserved nucleic acid, as a KD biomarker for various animals. We previously reported that altered levels of urinary exosome (UExo)-derived microRNAs indicate renal pathologies in dogs. This study comprehensively examined UExo-derived microRNAs, which reflected the KD status in cats. The examined cats were divided into two groups: normal renal function (NR) and KD. Based on our previous data in dogs and cats, as well as the present data on UExo-derived microRNAs in cats by next-generation sequencing, let-7b, let-7f, miR-10a, miR-10b, miR-21a, miR-22, miR-26a, miR-27b, miR-146a, miR-181a, miR-191, and miR-486a were identified as biomarker candidates. In summary, the levels of UExo-derived let-7b, miR-22, and miR-26a significantly decreased in cats with KD from the early stages of the disease. UExo-derived miRNA levels normalized to urinary creatinine or total RNA of miR-21a was significantly higher in the KD group. Importantly, the ratio of UExo-derived miR-21a to let-7b showed a significant and strongest correlation with serum creatinine (ρ = 0.751), blood urea nitrogen (ρ = 0.754), and urinary creatinine (ρ = −0.421) among all examined indices. Further, the ratio of miR-181a to let-7b or miR-10b significantly correlated with the progression of renal dysfunction in the KD group. Thus, we identified that UExo-derived microRNAs in cats, and their raw and normalized levels could indicate altered renal function.

## Introduction

The increase in the number of patients with various kidney diseases (KDs) has caused a global health problem in humans. In Japan, there are 13.3 million patients with chronic KD (CKD) (1). A similar increase in KD cases is occurring in aged companion-animals owing to the improvements in their maintenance conditions and advances in veterinary medical treatment (2). In particular, 30–60% of cats over 10 years of age showed CKD phenotypes (2, 3). Similar to human medicine, in veterinary medicine, blood urea nitrogen (BUN) and creatinine (CRE) are commonly used as renal function markers. In particular, blood CRE level is used to grade patients with CKD to different clinical stages as proposed by the International Renal Interest Society (IRIS), to ensure adequate therapeutic strategies (http://www.iris-kidney.com/guidelines/staging.html) in dogs and cats. For cats, IRIS staging of CKD based on blood CRE level is as follows: Stage 1 (<1.6 mg/dl); Stage 2 (1.6–2.8 mg/dl); Stage 3 (2.9–5.0 mg/dl); Stage 4 (>5.0 mg/dl). In recent basic and clinical studies, several biomarkers that manifest earlier than the elevation in blood CRE, such as the serum symmetric dimethylarginine, as described for human patients, were tested for the first time in dogs and cats (4). Cystatin C is also known to be a renal functional marker in humans and dogs; however, some researchers are skeptical about its application to cats (2). Basically, KD pathogenesis differ among companion-animals, and the glomerulus and tubulointerstitium tend to be injured in dogs and cats, respectively (5). Thus, although novel KD markers are crucial for the further development of veterinary medicine, species differences associated with molecular structures as well as renal function and pathogenesis, make it difficult to generalize and establish such markers.

We recently focused on the potential of microRNA (miRNA) as a novel disease marker by analyzing mouse KD models and companion animals (6–10), because miRNAs are stable small non-coding RNAs (18–25 bp) with high evolutionary sequence conservation among animal species. miRNAs can act as posttranscriptional regulators of target genes by binding to complementary sequences in specific target messenger RNAs (mRNAs) (8). Furthermore, recent studies revealed that several miRNAs are present in the 40–100 nm nano-sized exosomes in urine (8). Exosomes are a type of extracellular vesicles, such as microvesicles, apoptotic bodies, or ectosomes (8). Endosomal membrane budding of kidney cells results in the formation of multivesicular bodies (MVBs). The MVBs fuse to the plasma membrane which originates the urinary exosomes (UExos) (8). A recent study suggested that UExo-derived miRNAs could be a novel biomarker in KD. In particular, Khurana *et al*. reported miR-181a as the most robust and stable potential biomarker for human CKD (11). Furthermore, Li et al. reported that let-7c was significantly upregulated in UExos of diabetic nephropathy patients compared to that in the control group (12). Lv et al. reported that miR-29c and miR-21 levels in UExos correlated with renal fibrosis in human CKD patients (13, 14). Importantly, there is abundant evidence showing that miR-21 is a KD-associated miRNA, and it participates in renal fibrosis by targeting the transforming growth factor beta (TGF-β)/Smad signaling (15, 16). We also reported altered levels of miR-26a and miR-146a in the urine of CKD mice, that were mainly associated with glomerular and tubulointerstitial lesions, respectively (6, 7). In dogs, we analyzed UExo-derived miRNAs, especially miR-26a, which was significantly decreased in the KD group compared to that in the normal renal function (NR) group, and its decrease in the glomerulus was significantly correlated with the severity of glomerular injuries (9). Thus, the changes in UExo-derived miRNAs seem to be correlated with the progression of KD in various species. We previously reported the expression of miRNAs in the kidneys of dogs and cats by next-generation sequencing (NGS) (10). However, miRNA expression in feline UExos remained unclear, even though this species shows a high susceptibility to KDs. The use of UExo-derived miRNAs as KD biomarkers would be useful for the veterinary care of various species. Because some UExo-derived miRNAs originate from injured renal cells (17), they could be detected in liquid biopsies reflecting renal pathologies. This would represent an improvement over the classical biochemistry analyses using BUN and CRE, which are used to check for renal dysfunction.

In the present study, we examined miRNA candidates associated with changes in renal function in cats based on data obtained by NGS and KD-related miRNAs identified in previous studies.

## Materials and methods

### Ethics statement

The investigators adhered to the Guide for the Care and Use of Laboratory Animals of Hokkaido University, Faculty of Veterinary Medicine (approved by the Association for the Assessment and Accreditation of Laboratory Animal Care International). All sampling processes were carried out as part of clinical examination or diagnosis, and the owners of the animals provided informed consents. The present study retrospectively analyzed samples that were collected between 2012 and 2018.

### Animal patients

The cat urine samples were obtained from patients of the Veterinary Teaching Hospital, Hokkaido University (Sapporo, Japan) and Matsubara Animal Hospital (Osaka, Japan), and analyzed retrospectively. First, cats showing serum CRE (sCRE) levels over 1.6 mg/dL were selected as candidates having a risk of KD (IRIS; http://iris-kidney.com/guidelines/grading.html). Forty-five cats were diagnosed with KD based on sCRE, BUN, urine color, the diagnosis by a clinical veterinarian, and medical history from health record on the date of urine collection. KD samples showed ranges of serum BUN of 30.1–140.0 mg/dL and CRE of 1.8–14.0 mg/dL. Furthermore, based on sCRE levels, the KD group was divided into three groups: KD1 (1.6–2.8 mg/dL), KD2 (2.9–5.0 mg/dL), and KD3 (5.0 > mg/dL). Forty-three NR samples from individuals having a normal range of serum BUN (12.8–29.8 mg/dL) and CRE (0.4–1.5 mg/dL) were defined. All urine samples were stored at −30°C until further use. Urinary CRE (uCRE) was determined by Creatinine Companion assay kit (Exocell, Philadelphia, PA, USA).

### RNA purification from UExo-rich fraction

All urine samples (1 mL) were centrifuged at 2,000 × *g* for 30 min at 4°C to remove cells and debris. From the urine supernatant, total RNA from UExo was obtained using the 1-step column kit (Urine Exosome RNA Isolation Kit; Norgen; Thorold, ON, Canada) according to the manufacturer's instruction. All RNA solutions were adjusted to 100 μL with RNase-free water. The concentration of the obtained RNA was measured by NanoDrop 2000 (ThermoFisher Scientific; Waltham, MA, USA). The concentrations of the total urinary RNA (uRNA) are listed in Table 1. The average absorbance ratio 260/280 was 1.30 and 1.26 in NR and KD groups, respectively.

**Table 1:** Summary of renal function indices in cats.

			**sCRE** **(mg/dL)**	**BUN** **(mg/dL)**	**Age** **(Years)**	**BW** **(kg)**	**uRNA** **(ng/dL)**	**uCRE** **(mg/dL)**
	**Grouping criteria**		**Mean**					
NR	1.6<		1.1	21.9	7.6	4.6	7.3	217.9
KD1	1.6–2.8		2.3^***^	41.4^***^	11.6^*^	4.1	4.9^**^	97.8^**^
KD2	2.9–5.0		3.7^***^	60.9^***^	9.7	3.9	5.3^*^	151.1
KD3	5.0>		8.3^***^	122.7^***^	9.13	2.9^**^	7.3	114.9
KD	1.8>		3.9^***^	63.4^***^	10.4^*^	3.8^*^	5.5^**^	120.8^**^
	**Actual range**	***N***	***SD***					
NR	0.4–1.5	43	0.28	5.12	0.7	0.2	3.21	43.00
KD1	1.8–2.7	19	0.28	13.60	1.2	0.3	0.89	157.68
KD2	2.9–4.9	18	0.60	19.58	1.5	0.2	1.43	64.02
KD3	5.4–14.0	8	2.98	21.44	7.0	0.9	2.80	120.21
KD	1.8–14.0	45	2.49	33.87	0.9	0.2	1.77	89.23

NR, normal renal function group; KD, kidney disease group; sCRE, serum creatinine; BUN, blood urea nitrogen; BW, body weight; uRNA, urinary RNA; uCRE, urinary creatinine; SD, standard deviation; NR vs. KD1, KD2, KD3: Dunnett's test. NR vs. KD: Mann-Whitney U test.

* P < 0.05.

** P < 0.01.

*** P < 0.01

### NGS

NGS was carried out as previously reported (9, 10). Briefly, the quality of total RNA isolated from the UExo fraction by the column-based method (urine 1 mL) was checked using a Bioanalyzer (Agilent; Santa Clara, CA, USA), and the samples from NR and KD animals were pooled as one sample for each group. Total RNA (80 ng) obtained from each sample was used to construct sequencing libraries with the TruSeq Small RNA Library Prep Kit (Illumina; San Diego, CA, USA) according to the manufacturer's protocols. The quality of the libraries was assessed with the Agilent 2100 Bioanalyzer High Sensitivity DNA Kit (Agilent Technologies). The libraries pooled from the samples were sequenced using the Illumina HiSeq 1500 system (Illumina, San Diego, CA) in 51-base-pair (bp) single-end reads. Prior to the alignment to the reference genome, sequencing adaptors (mainly 3′ adaptors and contaminants of 5′ adaptors) and reads having too short inserts (<14 nt) or no inserts were trimmed with an in-house script. The trimmed reads were aligned to the mouse reference genome (mm 10) using the Bowtie 1.0.0 (18) software with the option –m 10,000, which allows reads to be aligned into multiple locations in the genome sequence. For quantification of the small RNA expression, the aligned reads were subjected to downstream analyses using StrandNGS 2.5 software (Agilent Technologies). The read counts allocated for the small RNA feature of mm 10 (version 2013.10.09) were quantified using a Trimmed Mean of M-value (TMM) method (19). The NGS data were deposited in the Gene Expression Omnibus database (United States National Center for Biotechnology Information) and are accessible through GEO Series accession number GSE121156 (https://www.ncbi.nlm.nih.gov/geo/query/acc.cgi?acc=GSE121156).

### miRNA analysis

We used 2.29 μL of RNA solution from 1 mL urine and 1.46 μL of reverse transcriptase solution of the TaqMan MicroRNA RT Kit (ThermoFisher Scientific) to produce complementary DNA (cDNA). The obtained cDNA solution (4.5 μL) was used for quantitative PCR (qPCR), qPCR analysis was performed using each miRNA-specific TaqMan primer (0.5 μL) and TaqMan Universal PCR Master Mix (4.5 μL, ThermoFisher Scientific) with an MX3000P system (Agilent; Santa Clara, CA, USA) and CFX Connect (Bio-Rad; Hercules, CA, USA). Mimic miRNAs (AccuTarget; Bioneer; Daejeon, Republic of Korea) were used to draw standard curves, and the net level of miRNA was calculated by a numerical formula. For normalizations, the obtained net level of miRNA was divided by the value of uCRE or that of the uRNA.

### Statistical analyses

Results are expressed as the median value. The Mann–Whitney *U* test was used to compare the two groups (*P* < 0.05). Kruskal-Wallis test was used for comparing over three populations or time points, and multiple comparisons were performed using Dunnett's test when a significant difference was observed (*P* < 0.05). Spearman's correlation test (*P* < 0.05) was used to analyze the correlation between the two parameters. Furthermore, for receiver operating characteristic (ROC) analysis, objective variables were examined as 0 (NR) and 1 (KD) by using JMP software (SAS; Cary, NC, USA). A stepwise regression analysis followed by binary logistic regression analysis was also performed (SAS; Cary, NC, USA).

## Results

### Clinical parameters of NR and KD cats

Based on sCRE, BUN, urine color, and the diagnosis by a clinical veterinarian, the obtained urine samples were divided into two groups: NR (*n* = 43, median = 7.4-years-old) and KD (*n* = 45, median = 10.5-years-old) (Table 1). Further, the KD group was subdivided into groups KD1-3, according to their sCRE levels. As shown in Table 1, KD cats showed significantly increased sCRE and BUN starting from the early disease group (KD1), compared to NR cats. Body weight, uRNA level, and uCRE were significantly decreased in KD cats compared to NR cats. Low body weight was apparent in KD3, and uRNA and uCRE were significantly decreased in KD1 or KD2.

Furthermore, as shown in Table 2, the majority of the analyzed samples were from mix-breed cats, in both the NR (58.1 %) and KD (42.2 %) groups. In both groups, the number of individuals that underwent castration or spaying was higher than that of intact cats.

**Table 2:** Summary of cat demographics and attributes.

**Group**	**Strain**	***n***	**%**	**Sex**	***n***	**%**
Normal renal function	Mix	25	58.1	Male	8	18.6
	Unknown	9	20.9	Castrated	21	48.8
	Persian (Chinchilla)	2	4.7	Female	3	7.0
	Abyssinian	1	2.3	Spayed	11	25.6
	American Shorthair	1	2.3		
	Maine Coon	1	2.3		
	Norwegian Forest Cat	1	2.3		
	Ocicat	1	2.3		
	Persian	1	2.3		
	Russian Blue	1	2.3		
	Total	43			43
Kidney disease	Mix	19	42.2	Male	12	26.7
	Unknown	9	20.0	Castrated	17	37.8
	American Shorthair	8	17.8	Female	6	13.3
	Norwegian Forest Cat	2	4.4	Spayed	10	22.2
	Persian (Chinchilla)	2	4.4		
	Abyssinian	1	2.2		
	Maine Coon	1	2.2		
	Russian Blue	1	2.2		
	Scottish Fold	1	2.2		
	Siamese	1	2.2		
	Total	45			45

### UExo-derived miRNAs determined by NGS in NR and KD cats

The result of NGS using UExo-derived RNAs is summarized in Table 3. After trimming procedures of NGS results, we obtained 19,701,266 and 21,499,030 reads from the NR and KD groups, respectively. The total number of reads corresponding to miRNAs were 18,704 and 23,874, respectively, and a total of 241 miRNAs were annotated as miRNAs. The percentage of detected miRNAs (0.16% in NR, 0.19% in KD) was quite low compared to other non-coding RNAs, similar to that reported for dog UExo-derived miRNA (9). Next, we selected the candidates as follows: (1) For down-regulated miRNAs in KD, miRNAs showing read number >150 in NR were selected; (2) For up-regulated miRNAs in KD, miRNAs showing read number >150 in KD were selected; (3) For fold change between the two groups, the miRNAs showing absolute values >2.0 were selected; (4) miRNAs expressed in the kidney were selected according to our previous study (10). Based on these criteria, we finally selected miR-486, miR-10b, miR-27b, let-7f, miR-26a, miR-146a, miR-21a, miR-181a, and miR-22 as biomarker candidates. miR-3107–5p was recently updated as miR-486b-5p, as they present the same mature sequence “uccuguacugagcugccccgag,” according to the mouse database (miRBase, http://www.mirbase.org/). Therefore, miR-3107 and miR-486a were considered to be the same mature miRNAs.

**Table 3:** Summary of results from next-generation sequencing targeting urinary exosome-derived RNA in cats.

**RNA**				**NR urine (%)**				**KD urine (%)**
miRNA				0.16				0.19
tRNA				89.98				87.50
Exonic RNA				0.65				0.45
Intronic RNA				0.85				0.72
Others				8.36				11.14
**Accession**	**Gene ID**	**NR urine** **(read number)**	**KD urine** **(read number)**	**Fold change** **(KD vs. NR)**	**Regulation** **(KD vs. NR)**	Kidney* (miR-X-3p)	Kidney* (miR-X-5p)	**Description**
miR-3107	MI0014103	261	1818	7.0	up	-	-	same as miR-486b-5p
miR-486	MI0003493	333	1225	3.7	up	-	+	miR-486a-5p, miR-486b-5p
miR-10b	MI0000221	7382	16319	2.2	up	+	–
miR-143	MI0000257	208	88	−2.4	down	–	–
miR-27b	MI0000142	427	171	−2.5	down	+	–
miR-30e	MI0000259	269	96	−2.8	down	–	–
miR-6236	MI0021583	256	90	−2.8	down	NA	NA
let-7f	MI0000563	239	71	−3.4	down	low	+
miR-26a	MI0000706	179	51	−3.5	down	low	+
miR-146a	MI0000170	242	62	−3.9	down	-	-
miR-21a	MI0000569	605	145	−4.2	down	-	-
miR-92a	MI0000719	234	52	−4.5	down	-	-
miR-181a	MI0000697	398	66	−6.0	down	low	+
miR-22	MI0000570	1275	160	−8.0	down	+	low
miR-378a	MI0000795	501	49	−10.2	down	–	–
miR-142	MI0000167	1758	130	−13.5	down	low	low

NR, normal renal function group; KD, kidney disease group.

A total of 241 genes were annotated as miRNAs. NR: normal renal function (n = 43) KD: kidney disease (n = 45). After trimming procedures in NGS, 19,701,266, and 21,499,030 reads were obtained from NR and KD groups, respectively. A total read numbers of 18,704 and 23,874 corresponded to miRNAs from NR and KD groups respectively. One pooled sample was analyzed in each group. Down-regulated genes in KD, genes showing read number > 150 in NR were selected. Up-regulated genes in KD, genes showing read number >150 in KD were selected. For fold change between the 2 groups, genes showing absolute values >2.0 were selected. ^*^Kidney expression information was referred from Ichii et al., Res Vet Sci. 2014, 96(2):299-303. -: not expressed. low: read number <4,000. +: read number >4,000. NA: not available.

### UExo-derived miRNAs determined by real-time PCR in NR and KD cats

**Figure 1:**
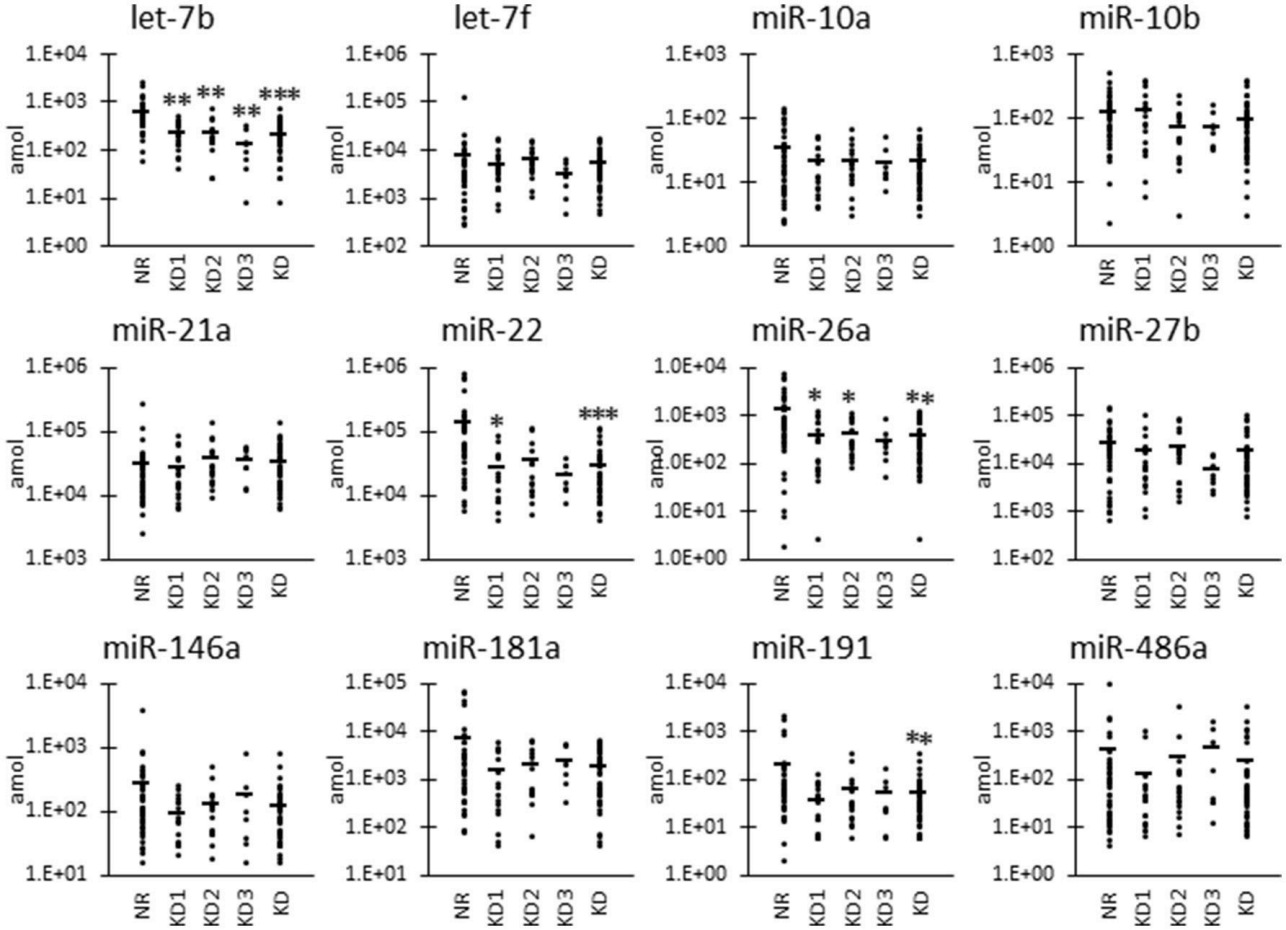
Raw miRNA levels in urinary exosome-derived miRNAs in cats. Cats were divided into the normal renal function group (NR, blood urea nitrogen (BUN) = 12.8–29.8 mg/dL, serum creatinine (sCRE) = 0.4–1.5 mg/dL, *n* = 43) and kidney disease group (KD, BUN = 30.1–140.0 mg/dL, sCRE = 1.8–14.0 mg/dL, *n* = 47). Furthermore, based on serum creatinine levels, the KD group was divided into three groups: KD1 (1.6–2.8 mg/dL), KD2 (2.9–5.0 mg/dL), and KD3 (5.0>mg/dL). Kruskal-Wallis test was used for comparing three populations or time points, and multiple comparisons were performed using Dunnett's test when a significant difference was observed (^*^, ^**^, ^***^ *P* < 0.05, 0.01, 0.001). TaqMan PCR method. Bars in graphs show the mean of each group.

In addition to the nine candidates presented in Table 3, we also included let-7b, miR-10a, and miR-191 in the quantification analysis, as important KD- or UExo-associated miRNAs based on previous studies (20, 21) and our canine study (9). Figure 1 shows the raw levels of UExo-derived miRNA in cats. As seen in the results, all miRNA levels tend to be decreased in KD compared to the NR group; except for miR-21a, a significant decrease was observed in let-7b, miR-22, miR-26a, and miR-191. Furthermore, let-7b, miR-22, and miR-26a were already significantly decreased in the KD1 group, which suggests that they are early-stage markers. miR-21a increased with disease progression, but a statistically significant change was not observed.

**Figure 2:**
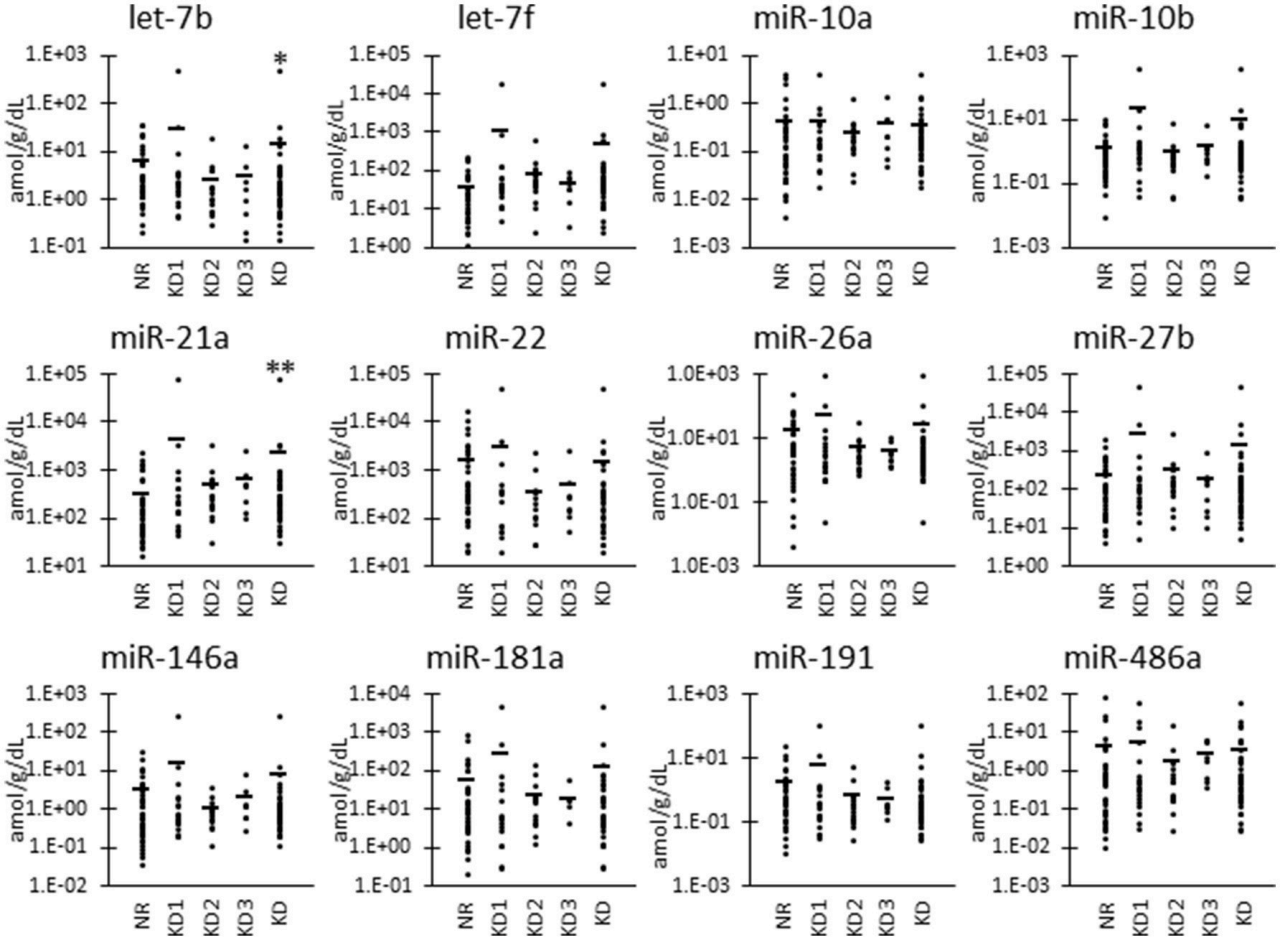
miRNA levels normalized to urinary creatinine level in urinary exosome-derived miRNA in cats. Cats were divided into the normal renal function group (NR, blood urea nitrogen (BUN) = 12.8–29.8 mg/dL, serum creatinine (sCRE) = 0.4–1.5 mg/dL, *n* = 43) and kidney disease group (KD, BUN = 30.1–140.0 mg/dL, sCRE = 1.8–14.0 mg/dL, *n* = 47). Furthermore, based on serum creatinine levels, the KD group was divided into three groups including KD1 (1.6–2.8 mg/dL), KD2 (2.9–5.0 mg/dL), and KD3 (5.0> mg/dL). Kruskal-Wallis test was used for comparing three populations or time points, and multiple comparisons were performed using Dunnett's test when a significant difference was observed (^*^, ^**^ *P* < 0.05, 0.01). TaqMan PCR method. Bars in graphs show the mean of each group.

As shown in Figure 2, we also examined the UExo-derived miRNA level normalized to uCRE. Normalized levels of let-7b and miR-21a were significantly decreased and increased, respectively, in KD compared to NR cats.

**Figure 3:**
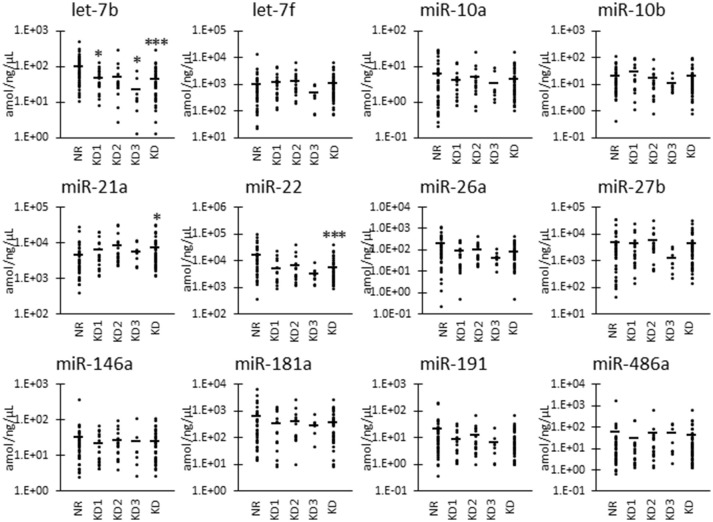
miRNA levels normalized to urinary total RNA level in urinary exosome-derived miRNA in cats. Cats were divided into the normal renal function group (NR, blood urea nitrogen (BUN) = 12.8–29.8 mg/dL, serum creatinine (sCRE) = 0.4–1.5 mg/dL, *n* = 43) and kidney disease group (KD, BUN = 30.1–140.0 mg/dL, sCRE = 1.8–14.0 mg/dL, *n* = 45). Furthermore, based on serum creatinine levels, the KD group was divided into three groups including KD1 (1.6–2.8 mg/dL), KD2 (2.9–5.0 mg/dL), and KD3 (5.0> mg/dL). Kruskal-Wallis test was used for comparing the three populations or time points, and multiple comparisons were performed using Dunnett's test when a significant difference was observed (^*^, ^***^: *P* < 0.05, 0.001). TaqMan PCR method. Bars in graphs show the mean of each group.

Furthermore, as shown in Figure 3, we also examined the UExo-derived miRNA levels normalized to uRNA. Normalized levels of let-7b and miR-22 were significantly decreased in KD compared to NR cats. miRNA let-7b showed a significant decrease in the KD1 group. However, miR-21a was significantly increased in KD compared to NR cats.

To analyze the specificity and sensitivity of the examined values for KD detection, we performed ROC analyses by defining the objective variables as 0 (NR) and KD (1) and explanatory variables as raw miRNA levels and miRNA normalized to uCRE or uRNA (Table 4). For raw miRNA levels, let-7b showed the highest area under the curve (AUC) with relatively higher specificity and sensitivity of the examined miRNAs. For normalized values by uCRE, miR-21a showed the highest AUC and tended to show higher specificity and sensitivity. For normalized values by uRNA, let-7b showed the highest AUC and tended to show higher specificity and sensitivity.

**Table 4:** Specificity and sensitivity of miRNA levels for KD detection.

		**AUC**	**Cutoff value**	**Specificity**	**Sensitivity**
Raw level	let-7b	0.848	226.948	0.881	0.705
	let-7f	0.458	18174.956	0.054	1.000
	miR-10a	0.545	52.933	0.225	0.953
	miR-10b	0.602	49.629	0.795	0.465
	miR-21a	0.601	12569.949	0.341	0.867
	miR-22	0.790	41155.995	0.730	0.811
	miR-26a	0.705	522.970	0.643	0.756
	miR-27b	0.572	20642.516	0.462	0.800
	miR-146a	0.621	186.592	0.357	0.867
	miR-191	0.583	36.166	0.732	0.600
	miR-181a	0.679	1306.982	0.625	0.564
	miR-486a	0.508	77.473	0.452	0.711
Normalized to uCRE	let-7b	0.393	499.161	1.000	0.023
	let-7f	0.680	28.635	0.700	0.698
	miR-10a	0.406	0.380	0.225	0.837
	miR-10b	0.563	0.285	0.400	0.791
	miR-21a	0.708	130.997	0.600	0.814
	miR-22	0.622	397.977	0.425	0.814
	miR-26a	0.455	0.484	0.200	0.977
	miR-27b	0.606	26.238	0.375	0.860
	miR-146a	0.557	0.462	0.475	0.814
	miR-191	0.490	0.119	0.275	0.837
	miR-181a	0.484	15.694	0.700	0.419
	miR-486a	0.412	19.726	0.075	0.977
Normalized to uRNA	let-7b	0.719	48.285	0.659	0.705
	let-7f	0.680	349.103	0.488	0.727
	miR-10a	0.483	5.610	0.341	0.795
	miR-10b	0.491	6.243	0.341	0.750
	miR-21a	0.641	2067.176	0.390	0.886
	miR-22	0.698	7954.128	0.585	0.818
	miR-26a	0.627	62.337	0.683	0.636
	miR-27b	0.488	2809.290	0.488	0.614
	miR-146a	0.540	26.718	0.415	0.727
	miR-191	0.568	6.458	0.610	0.591
	miR-181a	0.587	14.453	0.951	0.227
	miR-486a	0.443	15.877	0.366	0.659

*NR, normal renal function (n = 43); KD, kidney disease (n = 45); uCRE, urinary creatinine; uRNA, total urinary RNA; Objective variables were 0 (NR) and 1 (KD). AUC, area under the curve in receiver operating characteristic (ROC) curve*.

### Correlations between UExo-derived miRNAs and renal function in NR and KD cats

Table 4 summarizes the correlation between raw levels of UExo-derived miRNA and its levels normalized to uCRE or uRNA, and the renal function indices including BUN, sCRE, and uCRE. The ratio of each miRNA level to the other miRNA levels was also comprehensively analyzed. Data obtained by qPCR were used in this correlation analysis. As shown in Table 4, the raw levels and the levels normalized to uRNA, of let-7b and miR-22 showed a significant negative correlation with BUN and sCRE. The raw levels of miR-26a and miR-191 also showed a significant negative correlation with both BUN and sCRE. However, miR-21a levels normalized to uCRE showed a positive correlation with both BUN and sCRE. For uCRE, only raw level of let-7b showed a weakly significant negative correlation. Interestingly, in the miRNA ratio examined, miR-21a/let-7b and miR-21a/miR-22 (or their inverse number) showed a strong correlation with renal function. miR-21a/let-7b, as well as miR-181a/let-7b, and miR-181a/miR-10b showed a significant positive correlation with BUN and sCRE when we analyzed only the KD groups, suggesting their association with KD progression. For uCRE in KD groups, only miR-26a/miR-191 showed a significant correlation.

**Figure 4:**
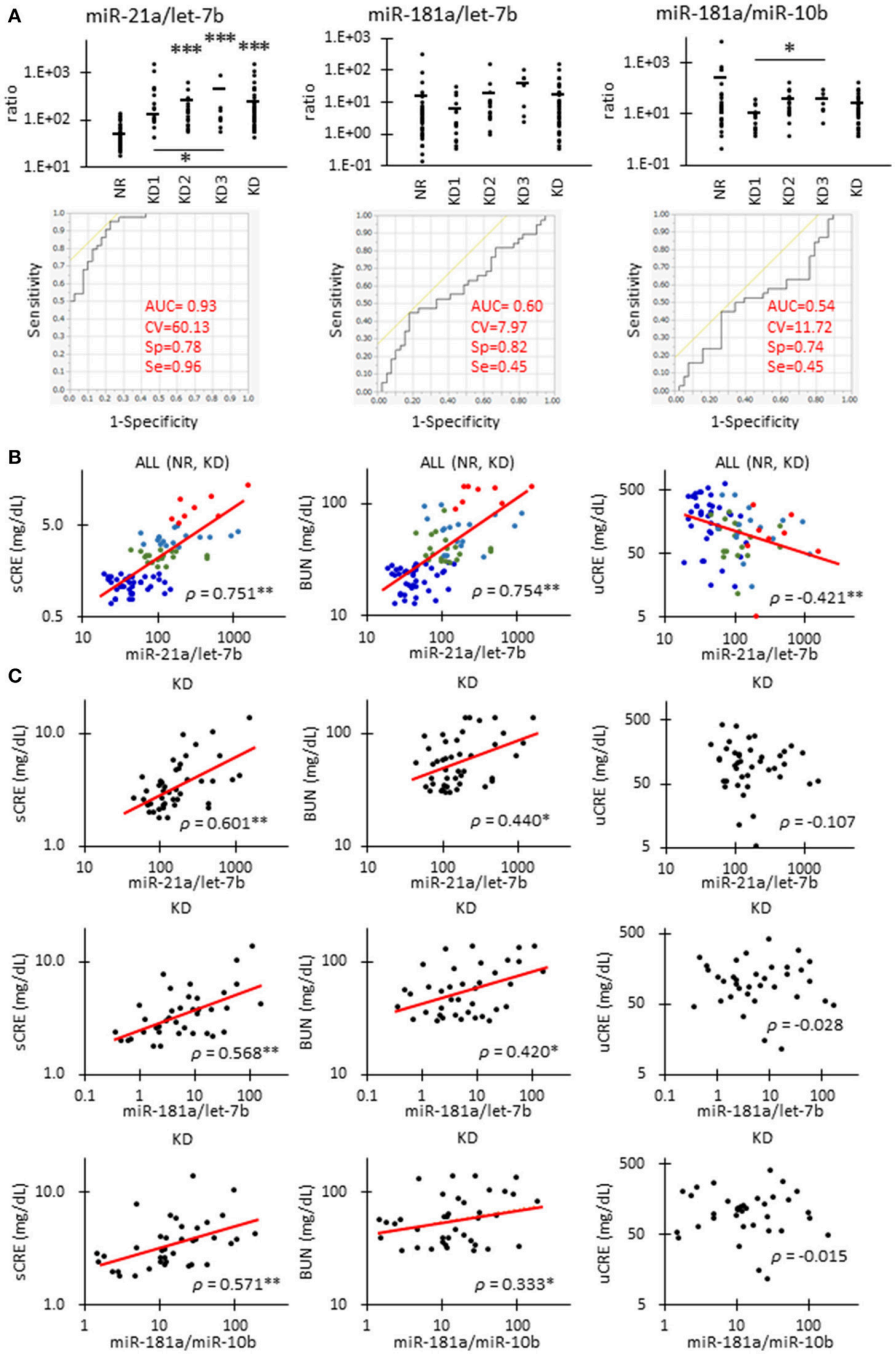
Correlations between miRNA parameters and renal function in cats. **(A)**The ratio of miR-21 to let-7b, miR-181a to let-7b, and miR-181a to miR-10b in urinary exosome-derived miRNA in cats. Cats were divided into the normal renal function group (NR, blood urea nitrogen (BUN) = 12.8–29.8 mg/dL, serum creatinine (sCRE) = 0.4–1.5 mg/dL, *n* = 43) and kidney disease group (KD, BUN = 30.1–140.0 mg/dL, sCRE = 1.8–14.0 mg/dL, *n* = 47). Furthermore, based on serum creatinine levels, the KD group was divided into three groups including KD1 (1.6–2.8 mg/dL), KD2 (2.9–5.0 mg/dL), and KD3 (5.0> mg/dL). Kruskal-Wallis test was used for comparing over three populations or time points, and multiple comparisons were performed using Dunnett's test when a significant difference was observed (^*^, ^**^, ^***^ *P* < 0.05, 0.01, 0.001). TaqMan PCR method. Bars in graphs show the mean of each group. For each value, a receiver operating characteristic (ROC) curve was drawn. AUC, area under the curve; CV, cutoff value; Sp, specificity; Se, sensitivity. Objective variables were examined as 0 (NR) and 1 (KD). **(B)**Correlation between the ratio of miR-21 to let-7b and sCRE, BUN, and uCRE. Spearman's correlation test was used to analyze the correlation between the two parameters (^**^P < 0.01). NR (blue, *n* = 43), KD1 (green, *n* = 19), KD2 (light blue, *n* = 18), and KD3 (red, *n* = 8) cats were analyzed. **(C)**The correlation between the ratio of miR-21 to let-7b and sCRE, BUN, and uCRE. Spearman's correlation test was used to analyze the correlation between two parameters (^*^, ^**^ *P* < 0.05, *P* < 0.01). KD (45) cats were analyzed.

Figure 4 shows the correlations between renal function and candidate miRNAs showing the highest significance in Table 4. miR-21a/let-7b ratio was significantly increased in KD2 compared to NR cats (Figure 4A). miR-181a/let-7b ratio showed no significant difference among the groups, but the miR-181a/miR-10b ratio showed a significant difference between KD1 and KD3. Further, in the ROC analysis, miR-21a/let-7b showed a relatively higher sensitivity and specificity to detect KD compared to the other ratios and values examined and presented in Table 4.

The miR-21a/let-7b ratio significantly correlated with sCRE and BUN when we examined all the groups (Figure 4B, Table 4) or the KD group only (Figure 4C). miR-21a/let-7b ratio significantly correlated with uCRE when we examined all the groups (Figure 4B). Although miR-181a/let-7b ratio and miR-181a/miR-10b ratio showed no correlation with renal function when we examined all the groups, a significant correlation was observed with sCRE and BUN when we examined the KD group alone (Figure 4C, Table 5).

**Table 5:** Correlations between urinary exosome-derived miRNAs and renal function indices.

**Groups**	**Correction factors**	**Rank**	**BUN**		**sCRE**		**uCRE**	
All (NR, KD)	Raw, uRNA, uCRE	1	let-7b	−0.589**	let-7b	−0.596**	let-7b	0.230*
		2	let-7b/uRNA	−0.470**	miR-22	−0.482**	NA	
		3	miR-22	−0.460**	let-7b/uRNA	−0.460**	NA	
		4	miR-22/uRNA	−0.425**	miR-22/uRNA	−0.438**	NA	
		5	miR-26a	−0.344**	miR-21a/uCRE	0.352**	NA	
		6	miR-21a/uCRE	0.341**	miR-26a	−0.295**	NA	
		7	miR-191	−0.291**	miR-191	−0.294**	NA	
		8	miR-10b	−0.286**	miR-191/uRNA	−0.246*	NA	
KD	Raw, uRNA, uCRE	1	NA		miR-181a	0.351*	NA	
All(NR, KD)	Ratio	1	miR-21a/let-7b	0.754**	miR-21a/let-7b	0.751**	let-7b/miR-21a	0.421**
		2	miR-21a/miR-22	0.557**	miR-21a/miR-22	0.614**	let-7b/miR-10a	0.332**
		3	let-7f/let-7b	0.522**	miR-10a/let-7b	0.547**	let-7b/miR-486	0.259*
		4	miR-486/miR-22	0.475**	let-7f/let-7b	0.524**	let-7b/miR-22	0.252*
		5	miR21a/miR-26a	0.471**	miR-21a/miR-191	0.473**	miR-27b/miR-10a	0.231*
KD	Ratio	1	miR-21a/let-7b	0.440*	miR-21a/let7b	0.601**	miR-26a/miR-191	0.318*
		2	miR-181a/let-7b	0.420*	miR-181a/miR-10b	0.571**	NA	
		3	miR-181a/miR-10b	0.333*	miR-181a/let-7b	0.568**	NA	
		4	NA		miR-181a/let-7f	0.505**	NA	
		5	NA		miR-181a/miR-10a	0.445**	NA	

NR, normal renal function group; KD, kidney disease group; sCRE, serum creatinine; BUN, blood urea nitrogen; uCRE, urinary creatinine; NA, not available.

* , ** Spearman's correlation test (P < 0.05, P < 0.01) was used to analyze the correlation between two parameters

Finally, we performed a stepwise regression analysis followed by a binary logistic regression analysis using miRNA levels, its normalized values, and its ratio (Table 6). Objective variables were set as 0 (NR) and 1 (KD). Among the examined regression models using miRNA levels, its normalized values, and its ratio, the miRNA ratio using let-7b/miR-21a, let-7f/miR-22, miR-26a/miR-10a, and miR-486/miR-191 showed the highest AUC (0.998) and contribution (0.924) and the lowest AICc (17.816) and BIC (27.671). Furthermore, the combinations of let-7b and let-7b/miR-21a increased the AUC (0.951) compared with each single value (Table 4, Figure 4A). The combinations of let-7b and the miRNA ratio using let-7b/miR-21a, miR-486/miR-191, and miR-27b/miR-22 showed more higher AUC (1.000) and contribution (1.000) and lower AICc (10.909) and BIC (21.383).

**Table 6:** Regression models to estimate KD by using miRNA-derived parameters in cats.

	**Raw level**		**Normalized to uCRE**		**Normalized to uRNA**		**Ratio**		**let-7b**, let-7b/miR-21a*		**let-7b, let-7b/miR-21a,** **ratio**	
Intercept	5.991		−0.372		−1.969		10.361		4.679		2280.474	
Prediction value	let-7b	−2.70E-02	let-7b	−1.529	let-7b	−0.140	let-7b/	−1564.320	let-7b	−0.003	let-7b	−3.535
							miR-21a				
	let-7f	6.80E-04	miR-21a	0.057	let-7f	0.006	let-7f/	70.384	let-7b/	−244.323	miR-486/	130.008
							miR-22		miR-21a		miR-191
	miR-21a	2.10E-04	miR-22	−0.018	miR-21a	0.004	miR-26a/	−0.052			let-7b/	−228195.714
							miR-10a				miR-21a
	miR-22	−5.70E-05	miR-26a	−0.065	miR-22	−0.001	miR-486/	1.175			miR-27b/	3176.557
							miR-191				miR-22
	miR-26a	−5.20E-03	miR-146a	−2.391	miR-26a	−0.028					
					miR-146a	−0.051					
					miR-181a	−0.011					
					miR-486a	0.027					
AUC		0.992		0.984		0.989		0.998		0.951		1.000
Contribution (R^2^)		0.835		0.724		0.851		0.924		0.583		1.000
AICc		28.672		44.841		38.004		17.816		54.754		10.909
BIC		40.500		58.249		57.587		27.671		61.746		21.383

NR, normal renal function (n = 43). KD, kidney disease (n = 45). Stepwise regression analysis followed by binary logistic regression analysis. Objective variables were 0 (NR) and 1 (KD). AUC, area under the curve in receiver operating characteristic (ROC) curve. AICc, Akaike information criterion. BIC, Bayesian information criterion. ^*^ let-7b and let-7b/miR-21a is directly analyzed by binary logistic regression analysis.

## Discussion

In this study, we focused on the correlation between levels of UExo-derived miRNAs and renal function in cats. UExos of experimental rodents including mice and rats (22), cows (23), and dogs (9) were reported. Recent human studies also focused on the potential of UExo as a biomarker for various KDs, including diabetic nephropathy (24), lupus nephritis (25), or glomerulosclerosis (26). UExos appear to have several membrane proteins derived from each component of the kidney structure, and the enveloped miRNAs in UExo were also investigated as potential biomarkers (8).

For the examined samples, the KD group showed a significantly higher age compared to the NR group. Although there are no data about the relation between UExo-derived miRNA levels and aging, aging is an important factor in developing KD, especially in cats. CKD phenotypes are apparent in 30 to 60% of cats over 10 years of age (2, 3). Therefore, aging contributes to the alteration of UExo-derived miRNA levels by changing the kidney structure or function.

In the present analysis of UExo in cats, the content of miRNA was quite low compared to that of transfer RNA (tRNA), as shown in Table 3. This is similar to our previous results in canine UExos (9). However, this is different from the data of a previous study using human urine, where the most abundant non-coding RNA in UExo was miRNA, and the content of tRNA was lower than that of miRNA (11). For the isolation of UExo-derived miRNAs, the human study used the combination method, based on ultracentrifuge and phenol chloroform isoamyl alcohol, but we used the column-based one-step method in our current and previous canine study (9). Therefore, the choice of UExo isolation method could strongly affect the purification process of UExo-derived miRNAs, rather than species differences between humans and dogs and cats.

Another possibility to explain the increased tRNA and relatively lower read number in our data, compared to those of the human study (11) is that cells lysis occurred when the urine was frozen, leading to sample contamination. However, the fresh urine samples would also show some lysed cells derived from the kidneys and urinary tracts, including the ureter and urinary bladder. Additionally, UExos, and miRNAs present in these structures are relatively stable when frozen.

Although UExo-derived from detached kidney cells or cell lysis would be a useful source to obtain miRNA biomarkers, urothelium contamination in the urine should be taken into consideration. To select the candidate miRNA in this study, we applied and modified the selection criteria of our previous study (10). However, to obtain more reliable data, we should exclude the miRNAs expressed in the urothelium. The possibility of urothelium derived-miRNA contamination could also be an obstacle for the normalization strategy of the obtained data. Mestdagh et al. (27) reported a useful normalization method using all the RNA expression data in the samples. A software to select the best housekeeping gene, such as geNorm finder, would also be helpful to normalize the data. However, even if we could normalize the data by these methods, the contamination of miRNA-derived from the urothelium would contribute to overestimating the total RNA amount. Therefore, future studies analyzing the miRNA expression profile of ureters and bladders are needed, since these data could help to determine which miRNAs are specific to the kidney.

From the NGS results, we detected and selected the UExo-derived miRNAs. In the previous study, we identified the differentially expressed UExo-derived miRNAs between NR and KD dogs, including miR-3107/miR-486a, miR-21a, miR-10a, and miR-10b (9). Curiously, the quantitative results of UExo-derived miRNAs were different between NGS and TaqMan PCR analysis in the present cat study, as well as in our previous dog study (9). This difference was considered to be due to the fact that NGS uses pooled samples and TaqMan PCR uses individual analysis. Specifically, the individual differences in the latter analysis strongly affected the quantification results. miR-3107/miR-486a, miR-21a, and miR-10b were identified as candidates in the analysis of UExo-derived miRNAs in this study, as well as in our canine study (9). In addition, the other miRNAs such as miR-142, miR-378a, or miR-22 was selected by NGS analysis in cats but not in dogs (9). In particular, miR-22 was selected as an equally expressed UExo-derived miRNA between NR and KD dogs (9). Thus, the different patterns of UExo-derived miRNAs between cats and dogs would reflect the species differences of UExo excretion from the kidney and/or other urinary organs.

Based on the NGS data obtained, in quantitative analysis of candidate miRNAs by TaqMan PCR analysis, we selected miRNAs for examination according to the following criteria: (1) differentially expressed between NR and KD, and (2) expressed in the cat kidney. As a result, we found that the raw level of UExo-derived miR-26a significantly decreased in KD compared to NR cats. miR-26a is considered to be mainly expressed in the glomerular podocytes, and its down-regulation was associated with the altered dynamics of podocyte functional markers, cytoskeletal molecules, and TGF-β/connective tissue growth factor pathway (7, 28). A decrease in miR-26a was also found in the glomeruli of human patients with IgA nephropathy and lupus nephritis, CKD dogs, diabetic model mice, and glomerulonephritis model mice (7, 9, 28). Furthermore, miR-22 selected by NGS analysis was also decreased in the UExo of KD compared to that of NR cats. Altered miR-22 expression was reported in several KDs, and it was upregulated in the kidney of diabetic mice (29) and rhabdomyolysis-induced acute kidney injury (AKI) mice (30) but downregulated in human renal cell carcinoma (31). Further, miR-22 regulates the synthesis of collagen IV and α-smooth muscle actin in tubulointerstitial fibrosis by targeting phosphatase and tensin homolog (PTEN) (32). Therefore, these altered levels of UExo-derived miRNAs would indicate the KD status in examined cats. Importantly, KD pathogenesis differs among companion-animals, as the glomerulus tends to be injured in dogs and the tubulointerstitium tends to be injured in cats (5). Therefore, although the function and biological characteristics of tubulointerstitial lesion-related miRNAs, including miR-21a and miR-22, would be similar among both species, its dynamics would more closely relate to KD pathogenesis in cats than in dogs.

miR-21a was also selected by NGS analysis of UExo in cats. In contrast to the decreased level of miR-26a and miR-22, the level of UExo-derived miR-21a, normalized to uCRE and uRNA, increased in KD compared to NR cats. miR-21a is one of the most validated KD-associated miRNAs, and its role differs in AKI and CKD. Briefly, a positive feedback loop between miR-21a and hypoxia-inducible factor 1 subunit alpha (HIF-1α)/2α is mediated by the PTEN/Akt/mTOR pathway, which is involved in the reduction of epithelial apoptosis caused by ischemia-reperfusion-induced AKI (33). However, miR-21a participates in the pathogenesis of CKD, such as renal fibrosis by targeting the TGF-β/Smad signaling (15, 16). The KD cats examined in this study were selected based on renal dysfunction. Therefore, the altered miR-21a levels in UExo would indicate the extent of renal injuries due to AKI and/or CKD that occurred in the individuals.

The level of UExo-derived let-7b in KD cats was significantly decreased in both raw and normalized data. We focused on the let-7 family as KD-associated miRNAs, because let-7a and let-7f were already reported as significantly down-regulated miRNAs in glomerulus with glomerulonephritis (7), and let-7g was slightly increased in the kidney of this mouse model (6). The present NGS also showed let-7f as a down-regulated miRNA in the UExo of KD cats, although a significant difference was not found in the TaqMan PCR analysis. Our previous studies did not report let-7b levels, even though it is known as an important KD-associated let-7 family member. Briefly, let-7b expression is reduced in the mouse models of renal fibrosis that upregulate TGF-β1 receptor 1 (TGFBR1), let-7b directly represses TGFBR1, and extracellular matrix proteins expression, it also decreases SMAD3 activity, and attenuates the profibrotic effects of TGF-β1 (21). Furthermore, some studies revealed that let-7b could be a serum marker for IgA nephropathy (20). UExo-derived let-7b was not examined in other animals, but we found a decreased pattern in KD cats, and this tendency may indicate the KD status of examined cats.

To take into consideration the urinary dilution due to KD status in cats, we measured the uCRE and uRNA; these were decreased in KD compared to NR cats. However, raw levels of all examined miRNAs, except for let-7b, did not significantly correlate with uCRE, indicating that the dilution of urine and UExo-derived miRNA levels did not completely correlate. The exosomal transfer is reported in cultured human renal proximal tubule cells to distal tubule and collecting duct cells (34). However, the UExo reabsorption in primitive urine was not fully clarified. From our results, the amounts of UExo-derived miRNAs were not strongly affected by urinary dilution. In regard to this result, the strongest correlation between renal function markers and UExo-derived parameters was observed in the ratio of miR-21a to let-7b, rather than any raw values or values normalized to uCRE or uRNA. Furthermore, as shown in Table 4, UExo-derived miR-21a and let-7b levels were strongly affected by the development of KD in cats, compared to other miRNAs. These results might indicate that the ratio of miR-21a to let-7b containing UExos, or the ratio of these miRNAs in the same UExo, could reflect the stage of renal injury. Interestingly, the raw and normalized levels of miR-181a and miR-10b were unchanged in the UExo of cats, but their ratio differed between NR and KD cats and correlated with the progression of renal dysfunction in KD groups. These data also indicate the importance of analysis focusing on the ratio of miRNA in UExo. Importantly, UExo-derived miR-181a was reported as the most robust and stable potential biomarker for human CKD (11). Further, UExo-derived miR-10b was significantly decreased in KD compared to non-affected dogs (9), and a human UExo study revealed that miR-10b was one of the most abundant urinary miRNAs (35). Therefore, we considered that the ratio of miR-21a to let-7b and miR-181a to miR-10b indicated the decrease of renal function in all examined cats and disease progression in KD cats. Especially, the ratio of miR-21a to let-7b showed the higher sensitivity and specificity to detect KD compared to the other values examined in the present study. By combining miR-21a/let-7b and the other ratios, we could estimate KD development. To increase the sensitivity and specificity for clinical levels, other miRNAs including cat-specific unknown miRNAs should be analyzed. These indicators might be able to detect individuals showing normal renal function parameters but abnormal renal histopathology.

In conclusion, we revealed the UExo-derived miRNA patterns in KD and NR cats and related them with several parameters indicating KD status. However, to address the limitations of the present study, we have to increase the miRNA purification yield and clarify the original cells of detected miRNAs for the future application of UExo-derived miRNAs to clinical veterinary medicine.

## Author contributions

OI, MH, TM, TN, TH, YHAE, and YK designed and performed the experiments and analyzed the data. HO, KM, KN, NS, MT, RS, and KO collected the clinical data and the samples. All authors were involved in writing the paper and approved the final manuscript.

### Conflict of interest statement

The authors declare that the research was conducted in the absence of any commercial or financial relationships that could be construed as a potential conflict of interest.

## Abbreviations

CKD: Chronic kidney disease
CRE: Creatinine
KD: Kidney disease
NGS: Next generation sequencing
NR: Normal renal function
ROC: Receiver operating characteristic
sCRE: Serum creatinine
uCRE: Urinary creatinine
UExo: Urinary exosome
uRNA: Urinary RNA.
