# Corrigendum: Urinary Exosome-Derived microRNAs Reflecting the Changes in Renal Function in Cats

**DOI:** 10.3389/fvets.2019.00002

**Published:** 2019-01-28

**Authors:** Osamu Ichii, Hiroshi Ohta, Taro Horino, Teppei Nakamura, Marina Hosotani, Tatsuya Mizoguchi, Keitaro Morishita, Kensuke Nakamura, Noboru Sasaki, Mitsuyoshi Takiguchi, Ryo Sato, Kazuhisa Oyamada, Yaser Hosny Ali Elewa, Yasuhiro Kon

**Affiliations:** ^1^Laboratory of Anatomy, Department of Basic Veterinary Sciences, Faculty of Veterinary Medicine, Hokkaido University, Sapporo, Japan; ^2^Laboratory of Veterinary Internal Medicine, Department of Veterinary Clinical Sciences, Faculty of Veterinary Medicine, Hokkaido University, Sapporo, Japan; ^3^Department of Endocrinology, Metabolism and Nephrology, Kochi Medical School, Kochi University, Nankoku, Japan; ^4^Section of Biological Safety Research, Chitose Laboratory, Japan Food Research Laboratories, Chitose, Japan; ^5^Veterinary Teaching Hospital, Faculty of Veterinary Medicine, Hokkaido University, Sapporo, Japan; ^6^Organization for Promotion of Tenure Track, University of Miyazaki, Miyazaki, Japan; ^7^Matsubara Animal Hospital, Matsubara, Japan; ^8^Department of Histology and Cytology, Faculty of Veterinary Medicine, Zagazig University, Zagazig, Egypt

**Keywords:** urinary exosome-derived miRNA, cats, kidney disease, biomarker, next-generation sequencing

An author name was incorrectly spelled as “Kon Yasuhiro.” The correct spelling is “Yasuhiro Kon.”

The authors apologize for this error and state that this does not change the scientific conclusions of the article in any way. The original article has been updated.

